# Seroprevalence and associated risk factors of bovine neosporosis in the Khomas region of Namibia

**DOI:** 10.4102/ojvr.v90i1.2077

**Published:** 2023-04-05

**Authors:** Alaster Samkange, Simbarashe Chitanga, Georgina N. Tjipura-Zaire, Vimanuka G. Mutjavikua, Jan W. Smith, Luis Neves, Tshepo Matjila

**Affiliations:** 1 Department of Veterinary Tropical Diseases, Faculty of Veterinary Science, University of Pretoria, Pretoria, South Africa; 2 Department of Production Animal Clinical Studies, Faculty of Health Sciences and Veterinary Medicine, University of Namibia, Windhoek, Namibia; 3 Department of Veterinary Pre-Clinical, Faculty of Health Sciences and Veterinary Medicine, University of Namibia, Windhoek, Namibia; 4 Department of Biomedical Sciences, Faculty of Health Sciences, University of Zambia, Lusaka, Zambia; 5 School of Life Sciences, College of Agriculture, Engineering and Sciences, University of KwaZulu-Natal, Durban, South Africa; 6 Directorate of Veterinary Services, Windhoek, Namibia; 7 Centro de Biotecnologia, Universidade Eduardo Mondlane, Maputo, Mozambique

**Keywords:** seroprevalence, cows, risk factors, *N. caninum*, Khomas, Namibia

## Abstract

*Neospora caninum* is a coccidian parasite that occurs worldwide and is one of the most important causes of abortion, especially in cattle. However, no studies have been performed in Namibia to determine the *N. caninum* status in livestock. Therefore, this study aimed to determine the seroprevalence of *N. caninum* in cattle and the associated risk factors in the Khomas region of Namibia. A total of 736 sera were collected from cows in 32 farming establishments. These comprised 698 beef and 38 dairy cattle sera and were tested using a commercial enzyme-linked immunosorbent assay (ELISA) kit. Questionnaires were concurrently administered to determine possible risk factors associated with *N. caninum* seropositivity. A total of 42 sera were positive (all beef), giving an animal-level seroprevalence rate of 5.7%. Eight of the 32 establishments had at least one positive animal, giving a herd-level seroprevalence of 25%. There was no significant association between seropositivity and the presence of dogs, jackals, history of abortions, farm size, number of cattle or average annual rainfall. The establishments with moderate to high numbers of Feliformia were 9.8 times more likely to be seropositive to *N. caninum* than those with none to low levels of the former (*p* = 0.0245). The authors concluded that the seroprevalence level of *N. caninum* in the Khomas region was relatively low compared with other parts of the world and that the role of Feliformia in the epidemiology of bovine neosporosis needed to be further investigated.

**Contribution:**

Serological evidence of bovine neosporosis and the associated risk factors are reported in Namibia for the first time. This study contributes to the scientific body of knowledge on *N. caninum* in Africa, which is currently limited.

## Introduction

*Neospora caninum* is an obligate intracellular coccidian parasite of the Apicomplexa phylum and Toxoplasmatidae family, which occurs worldwide (Dubey et al. 1988; Gharekhani et al. 2021; Goodswen, Kennedy & Ellis 2013). The parasite primarily infects dogs and cattle as well as all major domestic livestock species, companion animals, chickens, sparrows, wildlife and captive animals, including deer, rhinoceros, rodents, rabbits, coyotes, dingos, wolves and foxes (Donahoe et al. 2015; Fereig & Nishikawa 2020; McAllister 2022). The canids are considered the definitive hosts, in which infection results in polyradiculoneuritis and polymyositis in young dogs, with dermatitis and neurological manifestations being characteristic in adult dogs (Decôme et al. 2019; Fereig & Nishikawa 2020).

Neosporosis is of significant economic importance in livestock, particularly cattle, in which infection is characterised by abortions, stillbirths, the birth of weak neonates, congenital malformation, increased numbers of culled cows and decreased milk yield (Gharekhani, Yakhchali & Berahmat 2020; Kierbić et al. 2019), with associated annual losses running into hundreds of millions of United States dollars (Demir, Eşki & Ütük 2020; Reichel et al. 2013). Infection in these intermediate hosts is through ingestion of food and water contaminated with sporulated oocyst (Dubey, Schares & Ortega-Mora 2007; McAllister 2022), with the subsequent vertical transmission in infected herds playing a more significant role (Gharekhani et al. 2020; Lefkaditis et al. 2020; Sinnott et al. 2017). Several risk factors have been identified to be associated with infection in livestock, and these include the presence and number of farm dogs (Dubey & Schares 2011), farm production systems and practices (Bartels et al. 2006; Dubey et al. 2007; Ghalmi et al. 2012; Otranto et al. 2003; Pare et al. 1998), antibodies against other pathogens such as bovine viral diarrhoea (Björkman et al. 2000), human population density (Schares et al. 2004) and the region within countries (Bartels et al. 2006).

While *N. caninum* is presumed to occur worldwide (Reichel, Wahl & Ellis 2020), there is wide variation in prevalence across countries and regions, with some countries not having any data. Within the southern African region, evidence of bovine neosporosis has been reported in South Africa and Zimbabwe, with varied prevalence (Adesiyun et al. 2020; Jardine & Last 1993, 1995; Jardine & Wells 1995; Njiro et al. 2011). Other hosts that have been reported to show evidence of infection in southern Africa include dogs (Jardine & Dubey 1992), birds (Lukášová et al. 2018) and wildlife (Seltmann et al. 2020). Despite the importance of the livestock sector in Namibia and the possible impact that *Neospora* infection can have on the productivity of this sector, there has been no study to determine the prevalence, distribution and potential risk factors associated with infection in commercial cattle production. Therefore, the purpose of this study was to fill this knowledge gap by determining the seroprevalence of *N. caninum* infection in cows and the associated risk factors.

## Materials and methods

### Study population and setting

**FIGURE 1:**
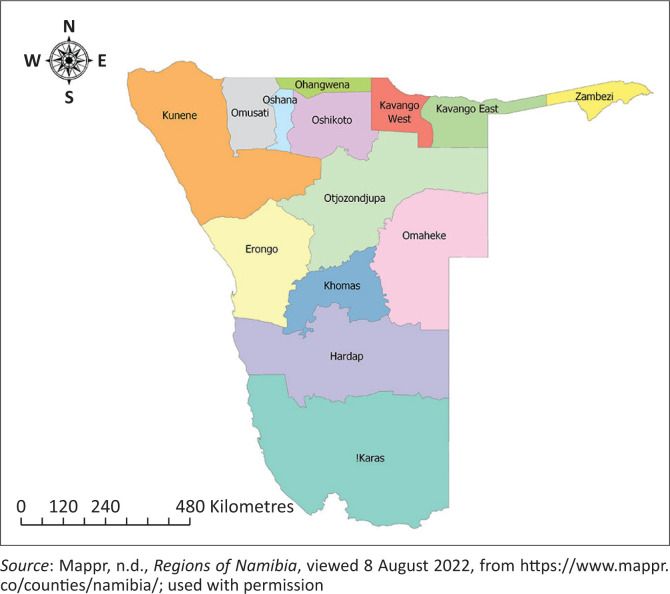
Map showing the 14 regions of Namibia in relation to the Khomas region, which lies in the central area of the country.

The study area was Namibia’s Khomas region, located in the central part of the country (Figure 1). Namibia’s sub-tropical climate varies from arid to semi-arid, and it is the driest country in sub-Saharan Africa (Mwazi & Shamathe 2007). The country’s central highlands receive an annual rainfall of between 300 mm and 400 mm and have an altitude of up to 1900 m (Kandiwa et al. 2017). The vegetation is predominantly shrub-veld and ambient temperatures range from 7 °C in winter to 33 °C in summer (Kandiwa et al. 2019). The Khomas region has about 556 farming establishments, with approximately 44 000 primarily commercial beef cattle (Directorate of Veterinary Services 2018). In addition, there are a few resettlement farms and communal settlements.

The study animals were the cows in the Khomas region of Namibia. The females were targeted because vertical transmission is the most important in the epidemiology of *N. caninum* (De Aquino Diniz et al. 2019; Dubey et al. 2007). In addition, Wei and co-workers found that female cattle had a higher seroprevalence rate than males (Wei et al. 2022). Therefore, targeting females increased the probability of detecting positive animals.

### Study design

This was a cross-sectional study design in which 32 farming establishments in the Khomas region of Namibia were selected. These comprised 26 commercial beef farms, three communal beef herds, two dairy, and one resettlement farm.

### Sampling and data collection

Sample size calculations were performed according to the methods by Pfeiffer (2002). For farm selection, estimated herd-level and individual animal-level prevalence rates of 20% and 10%, respectively, were used (Fereig et al. 2016; Nasir et al. 2012). A multistage sampling strategy was used to select the farming establishments included in this study. The Khomas region was first divided into clusters ranging from three to seven farming units. Seven clusters were chosen using convenience sampling, especially targeting farms that had previously reported abortions. Next, a stratified random sampling technique was used to select individual animals to be sampled at farm level, and a total of 736 cows were selected. A minimum of 10 cattle were sampled from each of the 32 farming establishments. A questionnaire was administered during the collection of serum samples to use that data to determine the possible risk factors of *N. caninum* in the Khomas region of Namibia.

Plain Vacutainer^®^ blood tubes and 20-gauge needles were used to collect blood from the coccygeal or jugular veins. Sera were extracted by centrifugation at 6000 revolutions per minute (rpm) for 10 min, after which they were stored at −20 °C until testing.

### Serological analysis

Indirect enzyme-linked immunosorbent assay (ELISA) (IDEXX Neospora X2^®^) (IDEXX Laboratories, Inc., Maine 04092, United States [US]) was used to detect specific anti-*N. caninum* immunoglobulin G (IgG) antibodies in the bovine sera according to the manufacturer’s instructions. The assays were duplicated, with absorbance values measured at 650 nm, with the sample to positive (S/P) ratio of 0.50 as the cut-off value (negative < 0.50 and positive > 0.50).

### Data analysis

The possible risk factors associated with *N. caninum* in sampled farming establishments were captured on questionnaires. The questionnaire and serology results were then captured in a Microsoft Excel 2013 spreadsheet. The captured data were analysed using descriptive statistics, chi-square test, odds ratios (ORs) and multiple regression analysis at a 95% confidence level. Regression analysis was used on quantitative data to analyse the relationship between the dependent variable (number of *N. caninum* seropositive cases per establishment) and the independent variables (the numbers of cattle and dogs per establishment, farm size and average annual rainfall). The chi-square test and ORs were used to analyse the relationship between categorical data (the history of abortions, sightings of stray dogs, number of jackals, number of Feliforma and rain scores over the previous three seasons) and the *N. caninum* status of each establishment. Finally, descriptive statistics were used to calculate the seroprevalence rates. All the statistical analyses were carried out using Microsoft Excel 2013.

## Results

**FIGURE 2:**
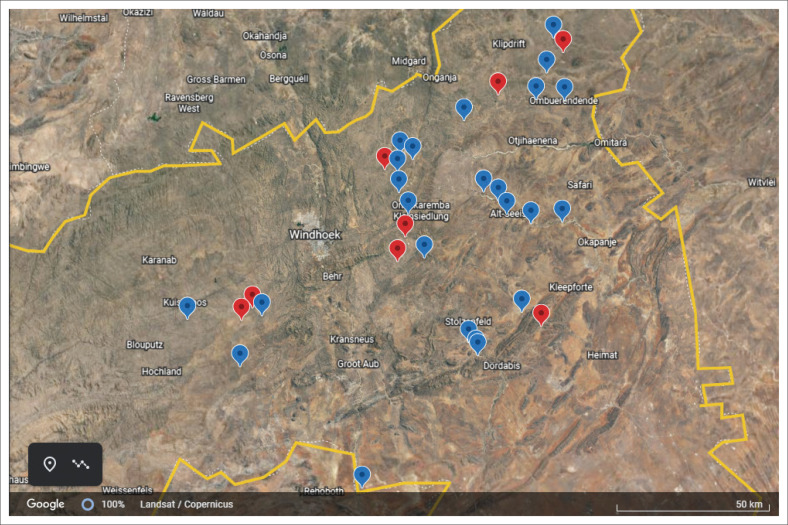
Google Earth map showing the distribution of the cattle farming establishments sampled in the Khomas region of Namibia, which is delineated in yellow. The eight positive farms are marked red, and the negative ones in blue.

In this study, 736 cattle (698 beef and 38 dairy) were sampled from 32 farming establishments, 30 of which were beef herds, and the remaining two were dairy. Eight of the 32 establishments had at least one positive animal, giving an overall herd-level prevalence of 25% (8/32), and they were widely distributed across the sampling sites (Figure 2). An overall animal-level prevalence of 5.71% (42/736) was observed, which varied widely across the farming establishments. For example, in the eight seropositive establishments, animal-level seroprevalence rates ranged from 3.03% (1/33) to 80.95% (17/21) (Table 1). The herd-level and animal-level seroprevalences for beef cattle only were 26.67% (8/30) and 6.02% (42/698), respectively. All the seropositive establishments were beef-producing commercial farms. The herd-level and animal-level seroprevalences for the two dairy farms sampled were 0% (0/2 & 0/38). In addition, all three communal establishments and one resettlement farm sampled (all beef) were seronegative.

**TABLE 1:** A summary of the *Neospora caninum* enzyme-linked immunosorbent assay test results for cattle sampled in the Khomas region of Namibia.

Farming establishment no.	Type of establishment	No. of cattle sera tested	No. of seropositive	Prevalence (%)
1	Beef commercial	12	0	0.00
2	Resettlement	32	0	0.00
3	Beef commercial	30	0	0.00
4	Beef commercial	18	0	0.00
5	Dairy	25	0	0.00
6	Beef commercial	39	0	0.00
7	Beef commercial	30	0	0.00
8	Beef commercial	43	3	6.98
9	Beef commercial	23	0	0.00
10	Beef commercial	33	1	3.03
11	Beef commercial	24	0	0.00
12	Beef commercial	40	0	0.00
13	Beef commercial	26	0	0.00
14	Beef commercial	25	0	0.00
15	Beef commercial	27	0	0.00
16	Dairy	13	0	0.00
17	Beef commercial	15	0	0.00
18	Beef commercial	25	0	0.00
19	Beef commercial	24	7	29.17
20	Beef commercial	18	0	0.00
21	Beef commercial	20	10	50.00
22	Beef commercial	21	17	80.95
23	Beef commercial	22	0	0.00
24	Beef commercial	20	2	10.00
25	Beef commercial	23	0	0.00
26	Communal	13	0	0.00
27	Communal	11	0	0.00
28	Communal	5	0	0.00
29	Beef commercial	18	1	5.56
30	Beef commercial	12	0	0.00
31	Beef commercial	26	1	3.85
32	Beef commercial	23	0	0.00

**Totals**	**-**	**736**	**42**	**5.71**

The results of possible risk factors for *N. caninum* seropositivity in cattle investigated using a questionnaire are shown in Table 2. The table summarises the results of the statistical analyses of the putative risk factors for seropositivity.

**TABLE 2:** A summary of the statistical analyses results performed on the possible risk factors associated with *Neospora caninum* seropositivity.

Possible risk factor	Analysis tool	*p*	Odds ratio	CI-intervals
Total no. of cattle	Multiple regression	0.946	-	−0.005 to 0.005
Number of dogs	Multiple regression	0.433	-	−0.255 to 0.112
Farm size	Multiple regression	0.713	-	−0.0003 to 0.0004
Average annual rainfall	Multiple regression	0.1428	-	−0.005 to 0.034
Number of Feliformia	Chi-square	0.024	9.8	0.061 to 4.504
Sighting of stray dogs	Chi-square	0.838	0.8	−1.769 to 1.435
History of abortions	Chi-square	0.497	0.7	−1.985 to 1.297
Number of jackals	Chi-square	0.854	0.6	−2.438 to 1.416

CI, confidence interval.

**FIGURE 3:**
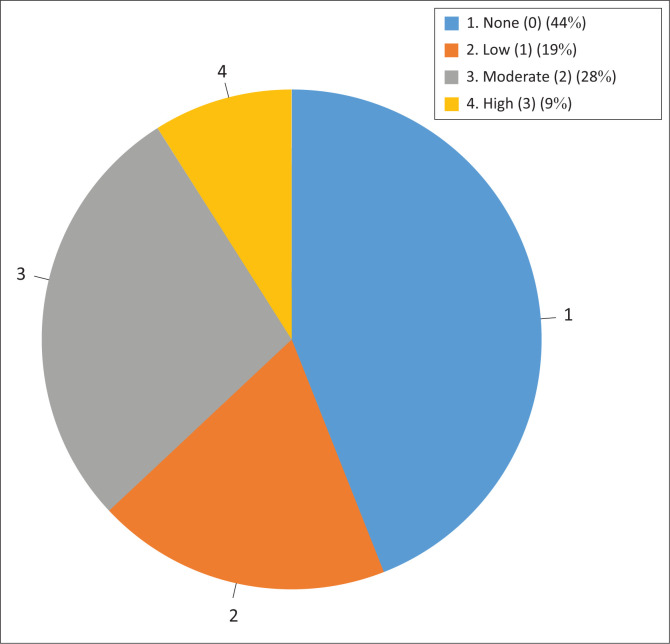
Distribution of abortion of scores among sampled establishments.

Eighteen of the 32 establishments (56.25%) sampled had a history of abortions during the previous 5 years, ranging from low levels of less than 10% (score 1), moderate levels of 10% to 15% (score 2) to at least one overt abortion outbreak (score 3) in the same period (Figure 3). Eleven of the 18 farms that reported at least one incident of abortions in the previous 5 years reported at least one incident of moderate to high abortion levels (scores between 2 & 3). Five of the eight farming establishments with at least one *N. caninum* seropositive animal had a history of previous abortions. However, this was not statistically significant on the chi-square test (*p* = 0.497).

**FIGURE 4:**
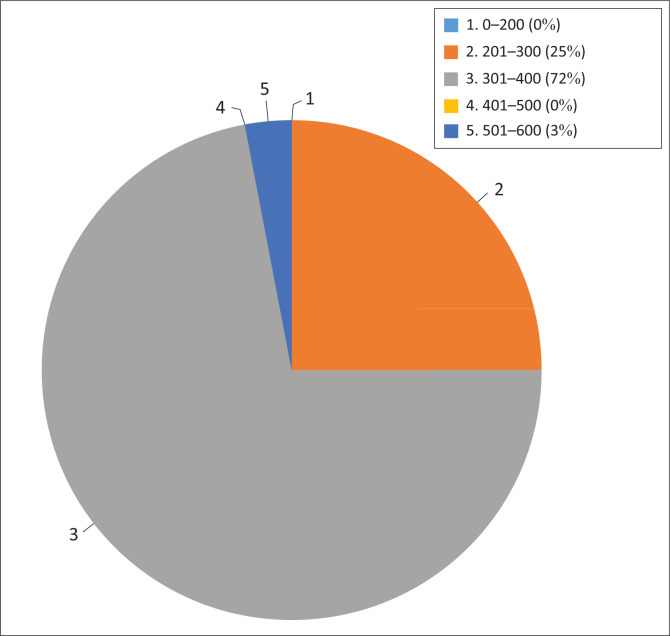
Average annual rainfall distribution among the sampled farming establishments.

The annual rainfall received per farming establishment averaged 344 mm per year and ranged from 224 mm to 600 mm annually. The average annual rainfall for 72% (23/32) of the establishments ranged between 301 mm and 400 mm per year, and 25% (8/32) of the establishments received 201 mm to 300 mm annually (Figure 4). However, the average rainfall per establishment was not significantly associated with *N. caninum* seropositivity on multiple regression analysis (*p* = 0.143).

**FIGURE 5:**
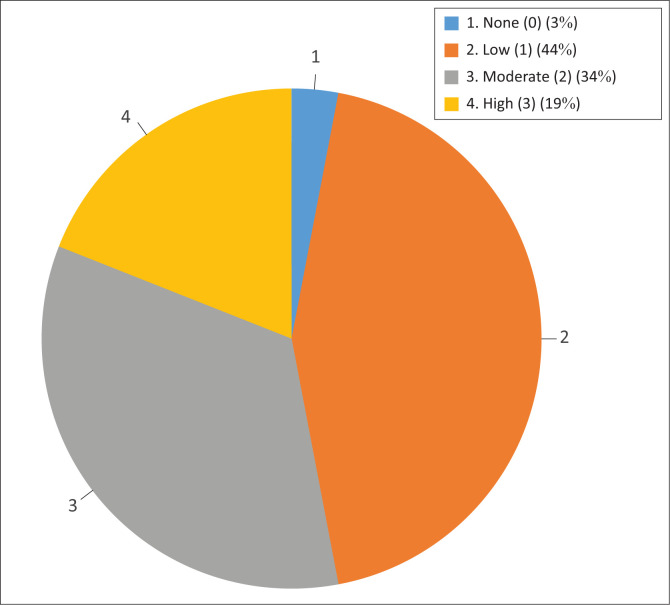
Feliformia score distribution among sampled establishments.

The sighting of Feliformia (primarily hyenas, cheetahs and leopards) at the farming establishments was significantly associated with *N. caninum* seropositivity among the cattle on the chi-square test and OR analysis. The establishments with moderate to high numbers of Feliformia on their properties were 9.8 times more likely (OR = 9.8; 95% confidence interval [CI]: 0.061 to 4.504) to be seropositive to *N. caninum* than those with none to low levels (*p* = 0.0245) (Figure 5).

**FIGURE 6:**
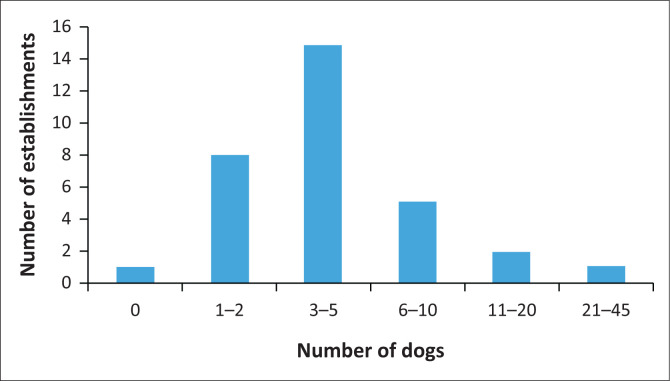
Distribution of dogs in sampled farming establishments.

Except for one, all the other 31 establishments had at least one dog, ranging in number from 1 to 41, with the latter being at a resettlement farm with many livestock owners and households (Figure 6). The average number of dogs per establishment sampled was six. The presence of dogs in the establishments was not significantly associated with seropositivity on multiple regression analysis (*p* = 0.433). On chi-square analysis, there was also no significant association between *N. caninum* seropositivity and sightings of stray dogs in sampled establishments (*p* = 0.838).

All the farming establishments reported observing high numbers of jackals on their properties (score 3); this was, however, not significantly associated with *N. caninum* seropositivity on chi-square analysis (*p* = 0.854).

**FIGURE 7:**
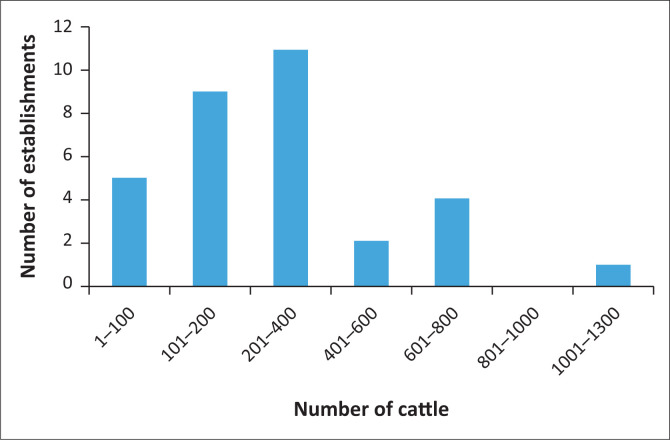
Distribution of the numbers of cattle in sampled cattle herds.

The average number of cattle per sampled establishment was 301, ranging from 13 to 1205 animals. The modal range was between 201 and 400 cattle (Figure 7). On multiple regression analysis, there was no significant association between cattle numbers per establishment and seropositivity (*p* = 0.946). There was also no significant association between farm size and seropositivity on multiple regression analysis (*p* = 0.713).

## Discussion

This study reports an overall animal-level seroprevalence of 5.7%, and to the best of the authors’ knowledge, this is the first study on *N. caninum* seroprevalence in cattle reported from Namibia. This observed prevalence is comparable to that reported within the region in South Africa, where 5.17% seroprevalence was reported in beef cattle (Chisi et al. 2013). However, unlike in the latter study, where all the dairy farms sampled were positive (Chisi et al. 2013), the two small-scale dairy herds sampled in this study were both negative. These results could be because the dairy industry in Namibia is still in its infancy, with an estimated herd of only 3000 cattle (Bieldt 2005). In addition, the largest dairy farm in the country was not sampled because it fell outside of the study area.

A similarly low-seroprevalence rate of 3.8% was also detected in one study in Iran, where it was attributed to a combination of warm and dry climate followed by cold and dry conditions (Noori et al. 2019). These climatic conditions were deemed unfavourable for the survival of *N. caninum* oocysts in the environment (Noori et al. 2019). On the other hand, humid conditions coupled with mild to warm environmental temperatures are ideal for the sporulation and survival of oocysts (Dubey et al. 2007) and have also been associated with higher incidences of *N. caninum* abortions (Wouda, Bartels & Moen 1999). Northwest China has a generally hot and dry climate with very little rainfall in the summer months compared with the other parts of the country, and the area has been found to have the lowest *N. caninum* seroprevalence rate of 9.4% in the country (Wei et al. 2022). These scenarios perfectly mirror the semi-arid conditions of the Khomas region of Namibia, where this study was conducted.

A study on cattle in northern Tanzania (Arusha region) determined a seroprevalence rate of 21.5% (Semango et al. 2019), which is much higher than the 5.7% found in this study. Given the semi-arid nature of Namibia’s climate, it could affect the survival and sporulation of the *N. caninum* oocysts in the environment (Dubey et al. 2007; Noori et al. 2019) and therefore reduce transmission. On the other hand, northern Tanzania is more humid and has higher average annual rainfall (Kimaro, Mor & Toribio. 2018) compared with Namibia, which provides a more conducive environment for the sporulation of *N. caninum* oocysts. The same argument could also explain the relatively low prevalence rate found in Namibia compared with other countries such as the northern part of Algeria (Ghalmi et al. 2012), Argentina (Moore et al. 2003), China (Qian et al. 2017), Colombia (Llano et al. 2018), North and Central America, Asia, Europe and India (Hebbar et al. 2022).

The low animal-level *N. caninum* seroprevalence rate in this study could also be attributed to the fact that 95% (698/736) of the samples tested were from beef cattle compared with only 5% (38/736) from dairy cattle. Beef cattle are less susceptible to *N. caninum* than dairy cattle, and therefore they tend to have lower seroprevalence rates (Fort et al. 2015; Gharekhani et al. 2020; Haddad, Dohoo & VanLeeuwen 2005; Quintanilla-Gozalo et al. 1999; Ribeiro et al. 2019).

Communal, dairy and resettlement establishments were all negative, possibly because the number of herds sampled from this category was very small; a larger sample size might have given a different result. Furthermore, feliforms are less likely to be found in this category of establishments because of higher human population densities. However, the dog population is also expected to be higher with the increased human population, especially in communal setups (Butler & Bingham 2000).

This study found no significant association between the number of dogs at the farming establishments and *N. caninum* seropositivity. Similar findings were also made in studies in Tanzania (Mathew 2017; Semango et al. 2019). However, as dogs are the definitive hosts of *N. caninum* (Goodswen et al. 2013; McAllister et al. 1998), these findings are somewhat surprising. Other studies have indeed confirmed that the presence of farm dogs increases the risk of *N. caninum* infection in cattle (Abdeltif et al. 2022; Dubey et al. 2007; Fávero et al. 2017; Robbe et al. 2016) and goats (Rodrigues et al. 2020), most likely through faecal contamination of pastures and open water sources with oocysts. The lack of significant association with the number of dogs found in the present study might have been caused by the fact that most of the farm dogs were confined to the homesteads and therefore did not have carte blanche access to livestock pastures. This, in turn, meant that the risk of pasture contamination, regardless of the number of dogs at the farming establishment, was markedly reduced.

In their study, Guimaraes and co-workers found a positive correlation in *N. caninum* seroprevalence between cattle and dogs (Guimaraes et al. 2004). Therefore, a low seroprevalence found in cows in this study could reflect the same scenario in farm dogs. However, such an inference can only be speculative because our study did not test the farm dogs.

The *N. caninum* positive status was significantly associated (*p* < 0.05) with the presence of Feliformia (brown hyenas, leopards and cheetahs) but not black-backed jackals, despite most farmers reporting many sightings of jackals on their properties. Domestic dogs (*Canis familiaris*) and black-backed jackals (*Canis mesomelas*) look physically similar; therefore, the possibility of mistaking these two species in places where the presence of Feliformia was reported cannot be entirely disregarded. In a recent Namibian study, brown hyenas (*Hyaena brunnea*) and black-backed jackals (*Canis mesomelas*) were found to be seropositive to *N. caninum* (Seltmann et al. 2020); however, the significance of these findings in light of the results of this study need to be further investigated. Furthermore, workers in Tanzania have also suggested the possible involvement of wildlife in the epidemiology of *N. caninum* after finding no association been dog ownership and cattle seropositivity (Semango et al. 2019).

In one study, self-rearing of replacement heifers was associated with an increased risk of bovine neosporosis (Otranto et al. 2003). However, this study found no such association, despite all the establishments indicating that they self-reared replacement heifers. This could be explained by the low *N. caninum* seroprevalence rate in the Khomas region, which reduces the risk of vertical parasite transmission.

This study found no significant association between abortion history and *N. caninum* seropositivity. One study in Northeast Algeria made similar findings, and the authors concluded that those cows were resistant to *N. caninum* abortions (Abdeltif et al. 2022). However, further investigations would be needed, given the extreme biological importance of the latter findings. The same scenario could also be at play in the Khomas region of Namibia, especially considering that significant variations in seropositivity have been found between countries, within countries, regions in the same country, and breeds (Dubey et al. 2007). Another study in Southeastern Iran found no association between *N. caninum* seropositivity and a history of abortions (Noori et al. 2019). However, other studies have demonstrated a significant association between *N. caninum* seropositivity and a history of abortions (Ghalmi et al. 2012; Llano et al. 2018; Moore et al. 2009). As noticed earlier, this discrepancy could be because of variations between countries or regions.

## Conclusion

The findings in this study were generally in agreement with some studies in other parts of the world. However, the seroprevalence level of *N. caninum* in the Khomas region is much lower than in most other parts of the world. Furthermore, the role of Feliformia in the epidemiology of bovine neosporosis needs to be further investigated.
